# Acute Calculous Cholecystitis Presenting as an Acute Abdomen in a Five-Month-Old Child

**DOI:** 10.7759/cureus.25180

**Published:** 2022-05-21

**Authors:** Estela Kakoo Brioso, Joana Jonet, Sofia M Antunes

**Affiliations:** 1 Pediatrics, Hospital De Cascais Dr. José De Almeida, Lisbon, PRT; 2 Pediatrics, Hospital de Cascais Dr. José de Almeida, Lisbon, PRT

**Keywords:** pediatrics, biliary calculus, acute abdomen, calcium bilirrubinate calculus, cholecystitis

## Abstract

Acute cholecystitis is an exceedingly rare condition in young children; nevertheless, it should be considered while investigating an acute abdomen. We report a case of a five-month-old male who presented to the pediatric emergency department with inconsolable crying, decreased oral intake, vomiting, diarrhea, and a tender right upper quadrant of the abdomen. Laboratory studies revealed elevated gamma-glutamyl transferase and alkaline phosphatase and the abdominal ultrasound was suggestive of acute calculous cholecystitis. The patient was treated with intravenous hydration and antimicrobial therapy, avoiding the need for emergent cholecystectomy. A calcium bilirubinate calculus was observed in the feces but no etiology was found after extensive investigation. We aim to raise awareness of this diagnosis and the need for prompt initiation of therapy to avoid complications.

## Introduction

An acute abdomen is defined as a sudden and severe abdominal pain associated with signs and symptoms suggesting a life-threatening condition that may require emergency surgical intervention [1]. In infants and toddlers, the differential diagnosis includes congenital anomalies, malrotation, hernias, Meckel diverticulum, intussusception, appendicitis, or infantile hypertrophic pyloric stenosis (IHPS) [2-4]. Acute cholecystitis is also a diagnosis to consider although exceedingly rare in children (3.76/100,000 persons) [5,6]. The clinical presentation and laboratory findings are nonspecific, and thus, ultrasound findings are essential for diagnosis.

We present a case of acute calculous cholecystitis in a five-month-old child. We aim to raise awareness of this diagnosis and the need to consider this as a possible differential diagnosis in this age group, despite its rarity.

## Case presentation

A five-month-old male presented to the pediatric emergency department with inconsolable crying, decreased oral intake, and vomiting since the previous day, accompanied by diarrhea (no blood or mucous in stools) since the day he was admitted. He had been medicated two days earlier with cefuroxime for suspected urinary tract infection (vomiting, no fever, and urine analysis with leukocyturia) with the transient improvement of symptoms. The urine culture came back negative for bacterial pathogens. There was no history of fever, jaundice, choluria, or acholia. The patient was otherwise healthy, exclusively breastfed, and thriving. The newborn screening for metabolic diseases, cystic fibrosis, and congenital hypothyroidism was negative. No relevant family history was present.

Physical exam showed an easily irritable and anicteric infant, with no signs of dehydration (despite the vomiting and decreased oral intake) but with a tender right upper quadrant of the abdomen upon palpation. Due to non-reassuring abdominal palpation, an abdominal ultrasound was performed.

**Figure 1:**
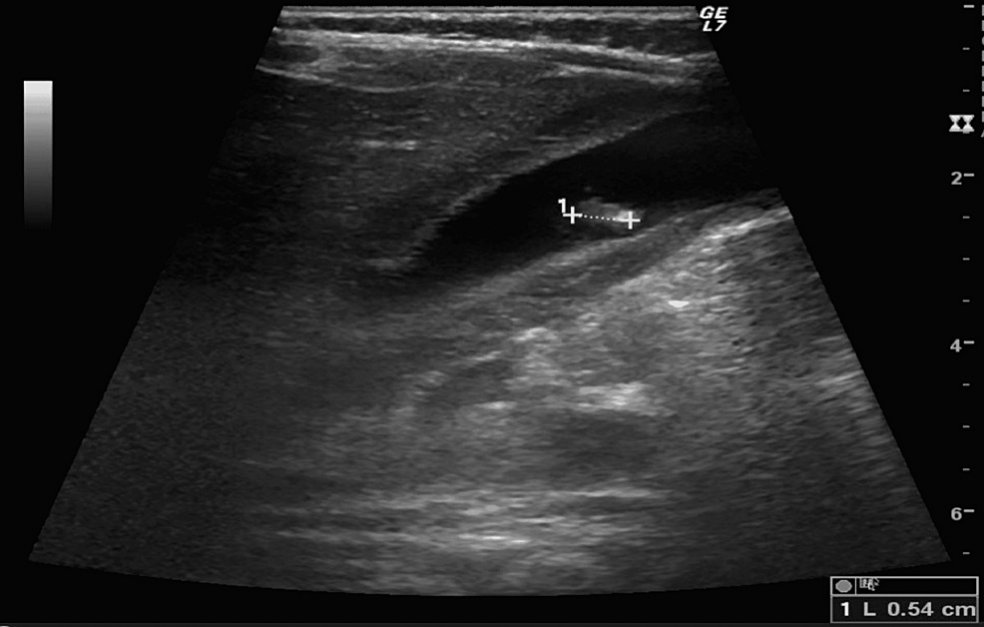
Admission abdominal ultrasound image demonstrating a distended gallbladder with thickened wall and an approximately 6 mm calculus.

There was no evidence of IHPS or intussusception but the ultrasound showed a distended gallbladder with thickened wall and a 6 mm calculus, suggesting acute calculous cholecystitis (Figure 1). Blood tests showed elevated gamma-glutamyl transferase (GGT, 136 IU/L), alkaline phosphatase (ALP, 183 IU/L), and lactate dehydrogenase (LDH, 323 IU/L), but normal total and conjugated bilirubin (0.16 mg/dL and 0.03 mg/dL, respectively), and aspartate (AST) and alanine (ALT) aminotransferases (39 IU/L and 60 IU/L, respectively) (Table 1). Inflammatory markers were within a normal range (normal white cell count and negative C-reactive protein [CRP] [0.23 mg/dL]), and there was no anemia nor elevated reticulocyte count.

**Table 1:** Biochemical results during hospitalization ALP: alkaline phosphatase; ALT: alanine aminotransferase; AST: aspartate aminotransferase; CRP: C-reactive protein; GGT: gamma-glutamyl transferase; LDH: lactate dehydrogenase

	Admission	3 days after admission	6 days after admission	8 days after admission
AST (IU/L)	39	189	66	46
ALT (IU/L)	60	343	181	80
ALP (IU/L)	183	281	267	217
GGT (IU/L)	136	480	387	276
Total Bilirubin (mg/dL)	0.16	-	-	0.25
LDH (IU/L)	323	292	-	-
CRP (mg/dL)	0.23	0.58	-	0.16

**Figure 2:**
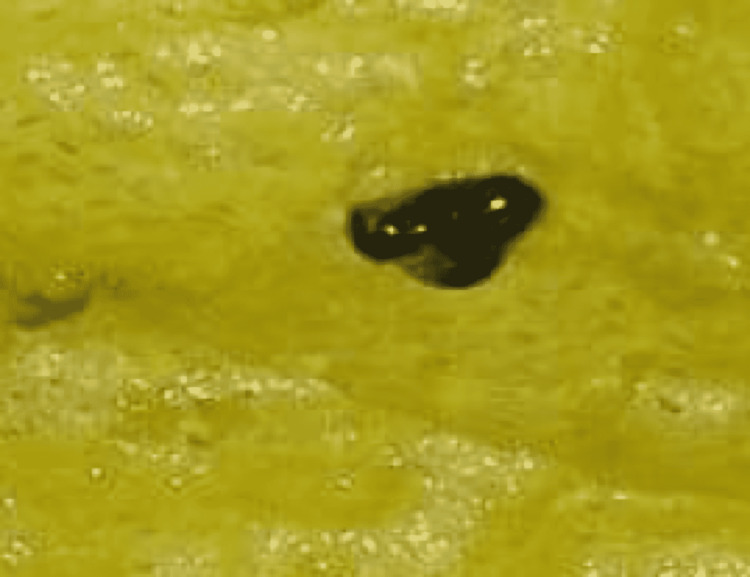
A dark and petrous calculus found in faeces on the third day of admission; laboratory analysis revealed a calcium bilirrubinate calculus.

After pediatric surgery consultation, he was admitted for clinical surveillance, intravenous (IV) hydration and antimicrobial therapy with IV ceftriaxone (first-line therapy according to the Tokyo Guidelines 2018), with no need for emergent cholecystectomy [7]. The patient gradually improved and became asymptomatic after three days, when a biliary calculus was observed in the feces (Figure 2). Subsequent analysis revealed a calcium bilirubinate calculus. Also on the third day of admission, the blood tests were repeated, showing an increase in almost all hepatic markers: AST 189 IU/L, ALT 343 IU/L, ALP 281 IU/L and GGT 480 IU/L (Table 1). No other changes were found: normal serum calcium levels (10.8 mg/dL), no anemia (hemoglobin 11.4 g/dL), and inflammatory markers were still within normal range (no leukocytosis, CRP 0.58 mg/dL).

On the fifth day of admission, the abdominal ultrasound was repeated, showing only biliary sludge, with partially distended gallbladder and no thickened wall. After consultation with the pediatric gastroenterology department, the patient was started on ursodeoxycholic acid (UDCA). The child completed a seven-day course of ceftriaxone and follow-up ultrasounds showed resolution of cholecystitis but resurgence of gallbladder stones: two more 6 mm calculi on the eighth day and only one 6 mm calculus on the twelfth day. Blood hepatic markers re-evaluations showed a consistent decrease and were, on the sixth and eighth days, respectively: AST 66 and 46 IU/L, ALT 181 and 80 IU/L, ALP 267 and 217 IU/L, GGT 387 and 276 UI/L, and total bilirubin remained normal (0.25 mg/dL on the eighth day) (Table 1). The lipid panel, collected eight days after admission, was normal.

The patient was discharged still on UDCA and with a referral for pediatrics, gastroenterology and surgery appointments for further investigation and follow-up. Extensive investigation did not find an etiology for the biliary calculus: hemoglobin electrophoresis, glucose-6-phosphate dehydrogenase, lipoproteins, bilirubin, calcium, fecal elastase, proteinogram and haptoglobin were normal. The blood smear showed moderate anisocytosis but no evidence of spherocytosis.

With more than two years of follow-up, repeated ultrasounds continue to show the 6 mm calculus in the gallbladder. The patient remains under outpatient surveillance and no other episodes of cholecystitis have since occurred.

## Discussion

The differential diagnosis for vomiting and right upper quadrant abdominal tenderness in a five-month-old child without fever must include IHPS, intussusception, and appendicitis. In our patient, these diagnoses were considered but discarded with the findings of the ultrasound. Acute cholecystitis is also a diagnosis to consider despite being exceedingly rare in young children [5,6].

The incidence of acute cholecystitis in the pediatric population is very low, with acalculous being the presentation in 30%-50% of cases, compared to 2%-17% in the adult population [6]. Acute calculous cholecystitis is the most common complication of gallbladder stones [8]. In children, gallbladder stones are usually secondary to chronic hemolysis (e.g. hemolytic anemia, hereditary spherocytosis), medication (e.g. cephalosporin treatment), total parenteral nutrition, cystic fibrosis, prematurity, metabolic diseases, and congenital heart disease, although frequently no predisposing condition is found [5,9,10]. Our patient was taking cefuroxime at the time of diagnosis but this is an unlikely predisposing factor since symptoms were already present before the start of the medication. Nevertheless, we cannot exclude that the IV treatment with ceftriaxone did not contribute to the formation of the new calculi. Due to the bilirubinate composition of the gallbladder stone, we considered hemolysis as a predisposing condition but no laboratory evidence of hemolysis was found. The hemoglobin electrophoresis and the blood smear were also not suggestive of a hemolytic disorder. Cystic fibrosis is also an unlikely diagnosis since the newborn screening was normal as was the fecal elastase concentration. Nonetheless, a genetic test and a sweat chloride measurement are needed to formally exclude this condition [11].

A patient with acute calculous cholecystitis usually presents with steady and severe right upper quadrant pain associated with nausea, vomiting, decreased oral intake, and fever. Additional investigation frequently shows an elevated white cell count, but abnormal liver function tests are infrequent [12]. In our patient, the transient elevation of liver function tests could have been caused by a bile tract obstruction that resolved with spontaneous stone expulsion. The diagnosis of acute cholecystitis requires the observation of gallbladder wall thickening or oedema on the abdominal ultrasound [12]. Definitive therapy is frequently accomplished with cholecystectomy but should also involve correction of the underlying process resulting in stone formation. Emergent surgery is indicated in patients with worsening symptoms despite medical therapy and hemodynamic instability. Our patient had neither and thus surgery was postponed. Medical therapy includes IV hydration, analgesic medication, and IV antibiotics to prevent complications such as gangrenous cholecystitis, perforation, and emphysematous cholecystitis [13]. The prognosis is variable and is mostly related to the timing of initiation of therapy and the cause of gallstone formation.

## Conclusions

Acute cholecystitis should always be considered early when investigating abdominal pain in infants and children to prevent complications, such as perforation and peritonitis, due to delay in therapy. Acute cholecystitis is easily diagnosed by ultrasound (a low-cost and non-invasive exam) but a high index of suspicion is needed in young children due to its rarity. Medical treatment with early IV fluids and antibiotics can avoid the need for emergent surgery.

In young children, an extensive investigation should be completed in order to exclude the multiple possible causes of gallstone formation. A multidisciplinary approach, with the early involvement of pediatric surgeons and pediatric gastroenterologists, is essential.
